# Impact of Magnetic Resonance Imaging on Healthcare in Low- and Middle-Income Countries

**DOI:** 10.7759/cureus.37698

**Published:** 2023-04-17

**Authors:** Bander S Hilabi, Sami A Alghamdi, Mansour Almanaa

**Affiliations:** 1 Radiological Sciences, King Saud University, Riyadh, SAU

**Keywords:** artificial intelligence in radiology, radiology, quality of care, magnetic resonance imaging, low- and middle-income countries (lmics)

## Abstract

Magnetic resonance imaging (MRI) played a significant role in the digital health platforms that influenced and supported modern medicine. However, there is a shortage of MRI in low- and middle-income countries (LMICs). The International Society of Radiology offers a detailed plan for LMICs to advance imaging quality in the global health agenda. The overarching objective of this scoping review was to determine the impact of MRI in healthcare in LMICs. This scoping review followed the Preferred Reporting Items for Systematic Reviews and Meta-Analyses guidelines to identify available evidence. We systematically searched four academic databases for peer-reviewed literature published between 2018 and 2021, namely, Medline, PubMed, Web of Science, and Scopus, as well as Google Scholar as a source for gray literature. The search identified 54 articles. We identified a range of reasons for introducing MRI in LMICs. Nonetheless, some challenges to accepting MRI as a method of healthcare have been reported, including technological, regulatory, and economical challenges. To implement the proposed plan, the involvement of professional and international organizations is considered crucial. The establishment of an International Commission on Medical Imaging under the umbrella of international organizations is suggested and collaboration with other diagnostic disciplines is encouraged to raise awareness of the importance of upscale diagnostics at large and to foster its integration into the care pathway globally.

## Introduction and background

Low- and middle-income countries (LMICs) are countries with a per-capita gross net income (GNI) between $1,036 and $4,045 and upper-middle-income economies/countries are those with a per-capita GNI between $4,046 and $12,535, with both considered to be developing countries. About 75% of the global population lives in middle-income countries [1]. Although magnetic resonance imaging (MRI) was invented in 1974, access to this innovative and clinically valuable imaging technique remains limited in developing/underdeveloped countries. Its usage in LMICs is still somewhat limited compared to the industrialized world, and it continues to advance each year exponentially due to stronger magnets.

Due to high acquisition costs, a lack of infrastructure, a continuous power supply, and the knowledge needed to maintain and operate the systems, MRI is generally restricted to developing nations [2-4]. The lack of specialists in MRI physics and clinical applications continues to be a significant problem for developing countries. Although the health indices are still poor, many developing nations have recently seen economic transition with better living standards, an aging population, increased healthcare knowledge, and access to cutting-edge medical treatments [5,6]. The issues with infrastructure, health service reform, and a lack of appropriately qualified personnel are addressed in that case. The changing healthcare landscape offers a market opportunity for MRI in LMICs [5,6]. For the diagnosis and treatment of many infectious diseases, such as tuberculosis [7], COVID-19 [8], and non-communicable diseases (NCDs), which are on the rise in LMICs, access to imaging services is essential. If existing obstacles are not addressed, it is unlikely that the sustainable development goals (SDGs), such as the idea of universal health coverage (UHC), set by health officials in many developing countries, will be met. The most effective methods for controlling disease in LMICs are primary prevention and risk reduction [9]. Imaging is crucial for primary prevention, detection, and effective treatment; therefore, equivocal access to MRI is a primary health right. We firmly believe that improvements are required to promote better access to and utilization of imaging in LMICs. To make MRI facilities more accessible in developing countries, it is imperative that training programs be generated and additional resources be deployed. This is because charging patients for an MRI at the same price as in industrialized nations would be improper. It is essential for the healthcare delivery systems in the developing world to improve access to MRI for advanced medical imaging. Therefore, having a solid grasp of the facilities’ current situation may serve as the foundation for planning and developing strategies to address the problems and seize any possibilities that might emerge soon. This review aims to discuss the difficulties that LMICs encounter when providing healthcare services, particularly MRI, and suggests ways to solve them.

## Review

Methodology

To identify the MRI facilities (government/public and private) available in LMICs, including the subregion of West Africa, a survey employing an online search was conducted from 2018 to 2021. Based on publicly accessible data, the number of MRI machines in West Africa and LMICs per million people was computed and compared to other regions of the world.

Goals and Context

A scoping literature review appears to be the most appropriate to produce insights for our research paper because of the wide scope of the study and the incorporation of all types of studies. The Preferred Reporting Items for Systematic Reviews and Meta-Analysis-Scoping Review (PRISMA-ScR) are followed in this study. The review process consisted of the following five iterative steps: (i) identifying the research; (ii) identifying relevant studies; (iii) selecting relevant studies; (iv) charting the data; and (v) summarizing outcomes.

Objective

This study aimed to evaluate the literature on the difficulties that LMICs encounter when providing healthcare services, particularly MRI, and to suggest ways to solve them.

Search Strategy

To identify possibly pertinent papers, we conducted searches in the following four electronic databases: Medline, PubMed, Web of Science, and Scopus. We incorporated peer-reviewed preprints from medRxiv due to the topic’s recentness. Google Scholar was used to look for gray literature, which provided information for the introduction and discussion sections. The three key principles of “MRI,” “healthcare,” and “low- and middle-income countries” served as the foundation for our search strategy.

Inclusion and Exclusion Criteria

We included studies published from 2018 to 2021 for a recent and reliable literature review. This paper included all nations classified by the World Bank as low-income (LICs), lower-middle-income (LMIs), or upper-middle-income countries (UMICs). Any type of medical intervention provided by hospitals, clinics, and healthcare professionals via MRI services that were accessible to patients and used for the diagnosis, treatment, and prevention of diseases and injuries was included in peer-reviewed studies if it was published in the English language.

Results

**Figure 1:**
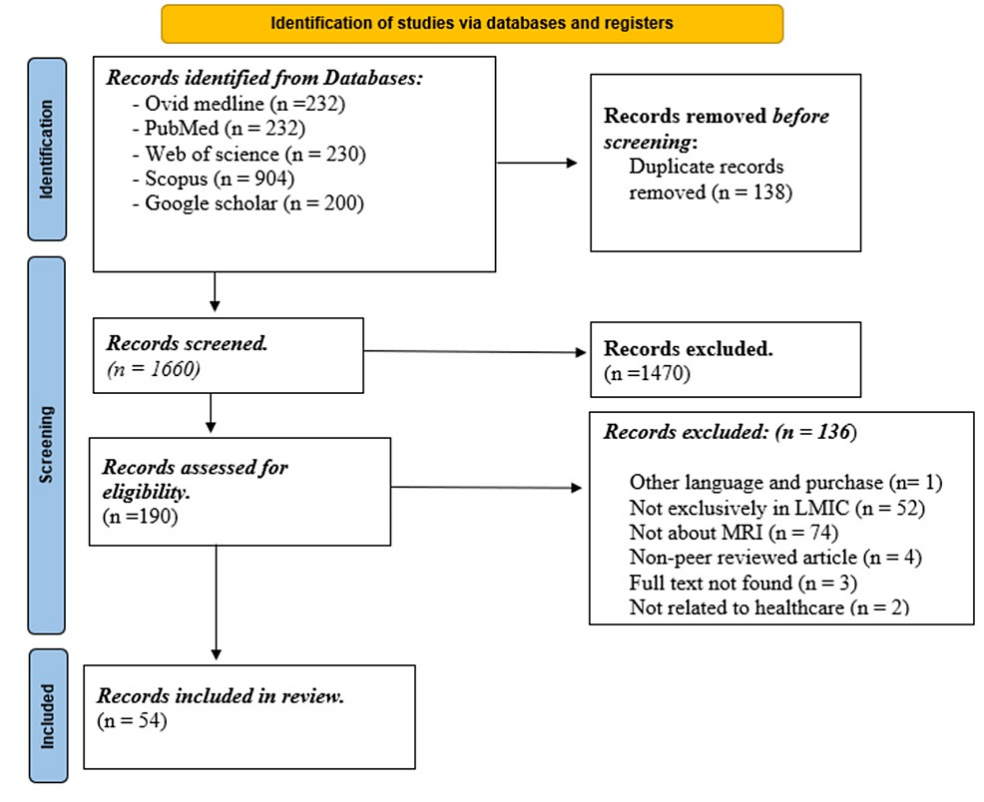
The flow diagram depicting the flow of information through the different phases of the systematic review. LMIC: low- and middle-income country; MRI: magnetic resonance imaging

Most studies were conducted in middle-income countries, with a quarter of the included studies (n = 13, 24%) conducted in India, 10 (18.5%) in China, seven (13.0%) in Brazil, and four (7.4%) in Turkey. Only one (1.9%) study was conducted in an LIC. Studies were included or excluded using the PRISMA guidelines (Figure 1).

Discussion

State of LMICs

Imaging has become essential in treating both contagious and non-contagious diseases. However, LMICs face severe personnel and imaging equipment shortages [10]. For MRI and nuclear medicine equipment, the gap is considerably more significant. Along with this equipment deficit, a severe labor shortage affects medical physicists, radiologists, and radiographers, with 1.9 versus 97.9 radiologists per million people in low versus high-income countries, respectively. The International Society of Radiology presents a comprehensive strategy for LMICs and provides a plan to increase the importance of imaging in the global health agenda.

Access to imaging services is essential for the diagnosis and treatment of many contagious diseases, such as tuberculosis [10], COVID-19, and other NCDs that are on the rise in LMICs [3]. The best methods for controlling disease in LMICs are recommended to be primary prevention and risk reduction [11]. However, because imaging is crucial for ensuring early disease detection and effective treatment, it would be unethical to deny LMICs access to the advantages that cutting-edge imaging offers affluent nations. Advancements are required to promote better access to and utilization of imaging in LMICs [12]. A summary of the LMIC situation is shown in Table 1.

**Table 1:** Key characteristics of included studies.

Characteristics	Number of studies (n)	Percentage (%) of all studies	Reported conclusions
Year of publication			-
2018–2020	30	55.6%	-
2020–2021	24	44.6%	-
Country			-
India	13	24.1%	Increased accessibility to healthcare. Reduced risk of infection
China	10	18.6%	Enhanced health outcomes
brazil	7	13.0%	Lack of technologist. Less risk of infection
Pakistan	3	5.6%	Financial and time saving
turkey	2	3.7%	Enhanced health outcomes
Libya	2	3.7%	Reduced need for multiple tests
Egypt	2	3.7%	Increased accessibility to healthcare
Mexico	2	3.7%	Enhanced health outcomes
The Philippines	2	3.7%	Financial saving
Lebanon	1	1.9%	More chances of early diagnosis
Peru	1	1.9%	Saving resources
Columbia	1	1.9%	Enhanced health outcomes
Kosovo	1	1.9%	Reduced risk of late diagnosis
Malawi	1	1.9%	Healthcare user satisfaction
North Macedonia	1	1.9%	Awareness of new medical technology
Ecuador	1	1.9%	Financial savings and better health outcomes
Iran	1	1.9%	Saving resources and better outcome
Sub-Saharan Africa	1	1.9%	Financial savings and better diagnosis

MRI Global Accessibility

Increasing access to and utilization of MRI services is essential to improving healthcare access and quality in Africa. MRI, first developed in the 1970s, has seen a meteoric rise in use as a primary diagnostic tool decades after its inception. This rapid expansion can be mainly attributed to the flexibility of MRI in detecting NCDs. However, despite a generally positive trend in the dissemination of MRI technology, its accessibility and use are limited in places with limited resources, such as Africa and Asia, due to high acquisition and maintenance costs, restricted access to infrastructure, a lack of expertise required to operate and maintain the equipment, and other region-specific factors [13,14]. More than 370 million people in West Africa were covered by only 84 MRI facilities in 2016, with more than two-thirds of those units located in Nigeria [15]. The African region had the lowest number of MRI scanners per million people in 2017 (0.7), followed by the South-East Asian region (1.1), the Eastern Mediterranean region (2.8), and the region of the Americas (4.1). The Asia Pacific region had the highest number of MRI scanners per million people in 2017 at 5.4. It is critical to comprehend and define medical device accessibility in light of the statistics on the density of MRI scanners worldwide and some of the technical difficulties mentioned above [16]. Particularly in Sub-Saharan Africa (SSA), where these services are even less accessible than they are in other parts of the continent, despite the critical role that MRI plays in the management of NCDs [7].

The availability of MRI scanners correlates favorably with internet use and socioeconomic status, showing the infrastructural needs and gaps for MRI access in developing areas [13]. A shortage of trained labor is particularly prevalent in SSA and the Indian subcontinent, where low scanner density is the norm [17]. This shortage exacerbates the difficulties with resource utilization. Additionally, MRI scanners must orchestrate several complex subsystems to provide diagnostically helpful images. Hardware design, manufacturing, and production are usually subject to strict restrictions because of their sophistication. This is associated with significantly increased system costs. For instance, the superconducting magnet’s uniformity substantially impacts the quality of the MRI images produced by a clinical scanner. To do this, the magnet must have longer or narrower bores. The bores have a detrimental effect on all five access dimensions and make up a significant portion of the system cost [18].

Numerous studies have been done on accessible healthcare [19-22]. Fortney and others identified the hallmarks of accessible healthcare in the digital era as five access variables (temporal, geographic, financial, cultural, and digital) at four levels of delivery. These access elements can be broken down into individual, health system, community, and provider characteristics. These statistics and access dimensions have been utilized in place of performance-based evaluation to review existing MRI systems according to accessibility [23], with further details shown in Table 2.

**Table 2:** Distribution of MRI units in selected countries globally (per million population).

Country	MRI units (approximately/million)	GDP
Japan (2014)	51.67	32,477
USA (2014)	38.96	56,116
South Korea	26.47	27,222
Spain (2014)	15.30	25,832
France	12.59	36,206
Portugal	9.90	19,222
Turkey (2014)	9.81	9,126
New Zealand	9.62	37,808
Canada	9.48	43,249
Qatar	9.22	73,653
Saudi Arabia	9.7	20,482
Mexico (2014)	2.25	9,008
Serbia	6.20	5,235
Libya	5.16	4,642
Israel	4.21	35,728
Uruguay	2.94	15,574
Tunisia	2.00	3,873
Morocco	0.36	3,004
Egypt	2.00	3,615
South Africa	2.90	5,724

Growing Role of Imaging

Imaging is essential to managing various disorders to ensure the continuum of care in the definition of the UHC concept, ranging from primary prevention, timely detection, and diagnosis to treatment and post-therapy rehabilitation or palliative care. Imaging plays a significant role in developing a precise and fast diagnosis, informing and guiding therapy choices, and enhancing treatment outcomes. Imaging is utilized to plan radiation procedures precisely, as well as to view various image-guided interventions in real time and to collect tumor samples for pathological analysis. A multi-society statement was recently released to clarify the value radiology offers to patients and healthcare, following the idea of value-based healthcare, a unique healthcare delivery paradigm [24]. Appropriate imaging is essential, especially in LMICs with scarce healthcare resources [25]. The European Society of Radiology is undertaking a project with the International Atomic Energy Agency (IAEA) to disseminate the ESR iGuide, ESR’s imaging referral guidelines embedded in clinical decision support [23], to improve the appropriate use of imaging in Africa.

Costs, Maintenance, and Precautions for Using MRI Machines

Imaging equipment that is expensive and technologically sophisticated cannot be used easily in LMICs, particularly in rural and semi-rural areas [26]. The ecosystem’s complexity explains why providing equipment to LMICs is challenging. Even in urban areas in LMICs, there is usually a shortage of dependable energy supply. Additionally, the need for well-equipped buildings with air conditioning and a special design for radiation protection is challenging [27]. Regular maintenance is needed, failure is common, and repairs are usually put off as they are typically done by specialist enterprises that serve a vast area. In addition, stringent requirements for radiation protection are frequently based on the International Basic Safety Standards (IAEA) [28], such as the call for the organization’s assistance at the national level, in addition to the availability of on-site medical physicists. When planning the operation of an imaging center for MRI, it is necessary to consider not only the upfront costs of purchasing equipment but also the ongoing costs of maintaining and operating such equipment over the long term. These costs constitute a sizeable amount of the overall costs [27]. Compared to MRI scanners, ultrasound devices often cost 10 times less and require significantly less maintenance than their more expensive counterparts [27,28].

Affordable MRI Usage

Because MRI technology is both expensive and complicated, there are few opportunities to use it in developing nations. This issue is being addressed by TU Delft and Leiden University Medical Center (LUMC) researchers, who are developing a less expensive and portable MRI scanner. A group of researchers from LUMC and TU Delft obtained money through Open Mind in 2016 for their study. They hope to use the scanner to make MRI scans for diagnosing hydrocephalus possible in less developed nations. However, a typical MRI machine is expensive and complicated. To run it, well-trained employees are required, and a technician must be sent out to fix it if something goes wrong [29]. That is not an issue in the Netherlands, but it is a different story if the machine is in Uganda. The answer is not a cheap, less effective MRI magnet and a standard computer to analyze the data, but rather the opposite, a more effective computer and a weaker, more affordable MRI magnet [30].

Barriers

Although widely used in high-income countries, imaging is not frequently used in LMICs for several reasons, such as lack of investment plans and prioritization. In LMICs, there are often insufficient investment plans and resources for procuring imaging technology. Furthermore, imaging requires much labor and capital, so it is frequently not prioritized in healthcare planning.

Equipment costs, maintenance, and safety:Expensive, high-tech imaging equipment is unsuitable for usage in LMIC environments, especially in rural and semi-rural locations. Even in urban areas, a reliable energy supply is frequently lacking, and it needs to be built in particular buildings with air conditioning and a unique design for radiation protection. Regular maintenance is necessary, failure is prevalent, and repairs are frequently put off because they are done by specialized businesses that typically serve a wide area. Additionally, there are stringent criteria for radiation protection (often based on the IAEA International Basic Safety Standards [31]), which need national organization and assistance and the availability of specialized professionals such as medical physicists. Nuclear medicine is subject to stricter regulations for the handling of non-sealed sources. This demonstrates that imaging involves more than just setting up medical equipment. It also involves a complex ecology that healthcare decision-makers are only dimly aware of. This may help explain why it is challenging to donate equipment to LMICs because the ecosystem’s complexity is not considered.

Although the expenses of purchasing equipment are substantial, long-term maintenance and operating costs comprise a sizable proportion of the total costs. They must be factored in when organizing the operation of an imaging center. On the websites of specialized associations for imaging equipment vendors, such as COCIR or DITTA, equipment costs are not made publicly accessible. Commercial websites can be used to shed light on the pricing range, even though there is a significant variety in the technological sophistication of the equipment [32]. In addition, maintenance and operating expenses are challenging to estimate due to the considerable local-specific variability. Compared to CT/MRI scanners, ultrasound machines are often 10 times less expensive and require less maintenance. Portable hand-held ultrasounds are affordable and perfect for expanding access to imaging in rural LMIC situations.

Operation of imaging equipment: A professionally qualified crew is required to operate radiology equipment, including radiologists, radiographers, and medical physicists who receive systematic training. Ultrasound is the most popular imaging modality in LMICs because of its affordability, ease of maintenance, and high practical yield. Compared to high-income countries, ultrasound training in LMICs is much less thorough and frequently modified to local conditions. A helpful strategy with shortened education of the limited personnel is necessary to increase its use. The significant income gaps between the commercial and public sectors worsen the public sector’s labor deficit. For LMICs, brain drain to the private sector or outside the nation is a significant problem [27].

Unraveling a Simply Convoluted Solution

The previously described complicated scene heavily revolves around utilizing a singular machine, a human operator, and a maintainer of the MRI machine. Artificial intelligence (AI)-assisted MRI software would solve this problem of scarcity. The software would leverage the potential of MRI images by providing the referring physician with immediate feedback by flagging the imaged cases as normal or abnormal. Thus, reducing the need for frequent human operators’ involvement and eliminating reliance on human resources. A casualty of utilizing the AI-assisted MRI models is increased quality of care by reducing missed or wrong diagnoses. Regarding using a single machine, it is, unfortunately, uncontrollable without committing to enormous resources that LMICs do not have. The last singular factor is the maintainer. The cost of maintaining an MRI machine is a massive hurdle for LMIC governments. Through AI-assisted modules, we can determine and predict maintenance checkpoints needed for each MRI machine with close to no downtime. It creates better efficiency and lifespan for the MRI machine and, therefore, better accessibility for the population. Governments and health institutions in LMICs can use little to nothing to leverage their limited resources to create better access to MRI machines [33].

Solutions to Remove the Obstacles

Technical remedies: Most recent advancements in MRI technology have been focused on digitalization, with standards for image preservation and transmission allowing for the sharing and pulling out of pictures and reports, as well as teleradiology interpretation services. This presents the opportunity to build networks as a foundation of the LMIC imaging organization. Additionally, teleradiology may be utilized to create complete imaging systems based on the network solutions between various levels of care, which will help compensate for the lack of skilled personnel. Modern, mobile MRI devices that run on batteries offer high-quality MRI at the point of care and can be utilized to answer various medical problems. By automating the detection of anomalies in the chest, brain, and other body parts, AI can enhance radiology workflow now and in the future, which might significantly have an impact [34]. Many AI software products are open source, and some businesses have AI built into their products. Free access to AI treatments for serious illnesses such as tuberculosis should also be encouraged. It has been anticipated that AI solutions will significantly affect imaging in LMICs with further research, improvement, and validation [35].

Legislative remedies: MRI can be used safely for staff and patients thanks to regulation. International organizations have created regulatory advice, such as the European Basic Safety Standards Directive and the International Basic Safety Standards, which may assist LMICs in introducing safety and quality in MRI through regulatory measures. However, clinical MRI practice varies significantly between and within nations, even when safety and quality regulations are in place [36]. The use of quality assurance programs, such as clinical audits, is advised to track compliance with regulatory standards. A foundation for quality and safety and quality controls is also necessary for teleradiology services [37].

AI-oriented solutions:The field of knowledge that creates computerized models to perform tasks that traditionally need human ability is known as AI. Machine learning (ML) is a branch of AI that trains computers to learn from experience, just like people and other animals do. ML uses algorithms to sift through massive volumes of data in search of patterns and extrapolations [38]. These algorithms are designed to recognize patterns in training data that they may apply to fresh datasets without being explicitly told to do so. By offering deep learning solutions for image capture, reconstruction, and analysis that eventually help clinical decision-making, AI is transforming the field of medical imaging, particularly MRI [39]. In several sequences, neural networks have recreated data from quickly captured underrepresented MRI images. Reconstruction of low-resolution 1.2 × 4.8 × 4.8 mm^3^ information captured in 50 seconds of scan time was made possible by a deep-learning-based, super-resolution MRI of the Angiography system [40,41]. A Multi-Scale Variational Neural Network under sampled reconstruction produced equivalent results. Utilizing a subset of convolutional neural networks, specifically the three-dimensional residual U-net, it is possible to perform super-resolution reconstruction of low-resolution, whole, three-dimensional, organ-balanced steady-state free precession (bSSFP) datasets. This can be done by employing the data. With high-resolution bSSFP of the whole heart, it will be possible to reach diagnostic accuracy and precision in patients [42]. In addition, AI-based imaging technology known as a virtual native enhancement can also be utilized in these areas. It does this by displaying native T1 mapping and cine imaging sequences as late gadolinium enhancement (LGE)-analogous pictures, which is made possible by using streams of CNN to utilize and enhance the acquired signal from those sequences. This approach enables contrast-free tissue characterization that is both efficient and effective, producing excellent agreement in the estimation of tissue burden and higher picture quality in comparison to the LGE images [43].

At this point, the manual outlining of picture contours by trained professionals is the usual clinical technique in MRI. However, this time-consuming process can result in variable results depending on the observer [37]. In adult populations, the segmentation of the right and left ventricles can be sped up using several different AI models that have been suggested and clinically validated. An automated segmentation for the quantitative measurement of tissue characterization for native T1 modeling in diagnosed cases with hypertrophic cardiomyopathy (HCM) has been established in a recent study using a deep, fully convolutional neural network. This segmentation shows robustness in interobserver variance and reduces time consumption to under a second [44]. Texture analysis (TA), a method that has recently gained popularity, uses various ML algorithms to measure the spatial variability and association of neighborhood pixels, which is necessary to compute advanced imaging metrics. It has been established that the texture features acquired from CMR offer the potential for further investigation and clinical implementation. The assumption is that the dispersion of pixel gray-level values represents meaningful information beyond the measured mean signal. TA features, generated through supervised ML models, have been demonstrated to differentiate and quantitatively evaluate the delicate discrepancies between two entities. For example, worldwide, T1 can distinguish HCM and hypertensive heart disease from regular hearts but not among the two as values overlap. This is even though these two parameters can distinguish HCM and hypertensive heart disease from normal hearts [45].

Because of the inherent soft-tissue contrast of MRI, a wide range of structural and physiological acquisition techniques, and its diagnostic potential, AI is used extensively in MRI. AI will usher in a new generation of quantitative imaging that will make full use of these massive data structures within the next few years, ushering in a period that will revolutionize MRI and transform its predominantly qualitative therapeutic applications. MRI, unlike any other modalities, contains a significant amount of information, and AI has all of the requirements to be the tool that could push traditional MRI scans to the next level for LMICs [46]. The introduction of these algorithms will help in the construction of multi-algorithm MRI and may help in device shortage in LMICs.

## Conclusions

Expensive equipment and commercial challenges, such as reimbursement and regulatory limits, are projected to ease. Improved socioeconomic situations in LMICs and government investments in healthcare infrastructure have increased demand for MRI. The role of AI-assisted MRI is, therefore, much more significant than ever. All tides are shifting toward more autonomous and accurate AI-assisted MRI models while utilizing limited resources. Focus and attention to technical developments is the optimal way to enhance the quality of care without wasting resources on expensive equipment and human resources.
